# Essential domains and sequences of the foamy virus Bet protein involved in binding and counteracting APOBEC3 restriction factors

**DOI:** 10.1186/1742-4690-10-S1-P49

**Published:** 2013-09-19

**Authors:** Dragana Slavkovic Lukic, Agnes Hotz-Wagenblatt, Martin Löchelt

**Affiliations:** 1 German Cancer Research Center DKFZ, Heidelberg, Germany

## Background

APOBEC3 (A3) cytidine deaminases restrict viral replication by efficiently editing viral genomes. To escape this restriction by lethal mutagenesis, lentiviruses have evolved the viral infectivity factor (Vif), which binds A3 proteins and targets them for proteolytic degradation. In contrast, foamy viruses (FVs) express high amounts of the accessory Bet protein allowing replication in the presence of A3, apparently by A3 binding/sequestration and thus preventing A3 packaging into virus particles. Due to virus-host coevolution, Bet can only counteract the activity of A3s from their cognate host species. FV bet genes appear to be ancient, since corresponding sequences are clearly present in endogenous FV sequences in different mammalian species.

## Materials and methods

Conserved motifs in Bet were identified by bioinformatics. Based on these findings, terminal deletion mutants, Bet chimera consisting of domains from distantly related FVs, and alanine scanning mutants of Bet were constructed and analysed using virological and cell biology methods.

## Results

Although FV Bet proteins are highly divergent, we identified six conserved motifs with a conserved spacing within the central and C-terminal parts of Bet encoded by bel2. In line with the localization of these conserved motifs within bel2, bel1 sequences were shown to be dispensable for basal A3 inactivation and A3 protein binding. To study the function of Bet motifs in detail, we created diverse targeted deletion and substitution mutants of feline FV (FFV), as well as chimeric proteins composed of prototype/primate FV (PFV) Bet and FFV Bet sequences. The potential of all mutants and chimeras to inactivate feline A3 (feA3) and physically bind to feA3 was analyzed. Bet proteins with substituted amino acids in the first, second and third conserved motifs were inactive in binding and counteracting feA3 proteins while mutations in the fifth conserved motif did not impair Bet function. Chimeric proteins composed of PFV Bel1 and full-length FFV Bel2 counteracted feA3-mediated restriction of FFV and physically bound to feA3. Studies performed with chimeric proteins also indicated that the last 22 amino acids of FFV Bet are not part of feA3Z2b binding and effector site.

## Conclusions

These results show that the Bel2 domain of Bet contains the A3 binding and effector sites and that the first three conserved motifs are essential for counteracting host-mediated restriction.

